# Association between nutritional status at admission and inadequate bowel preparation in inflammatory bowel disease patients: a retrospective cohort study

**DOI:** 10.3389/fnut.2026.1794988

**Published:** 2026-05-08

**Authors:** Fangxin Wei, Hongli Liu, Xiaomei Song, Tian Wang, Xiaoqin Tan, Haihua Zhang, Kailin Wang, Lu Gao, Yuan Yuan, Liming Wang, Hao Wang, Hong Guo, Jin Yu

**Affiliations:** Department of Gastroenterology, Chongqing General Hospital, Chongqing University, Chongqing, China

**Keywords:** bowel preparation, inflammatory bowel disease, nutritional risk index, nutritional risk screening, prognostic nutritional index

## Abstract

**Background:**

Malnutrition is highly prevalent among patients with inflammatory bowel disease (IBD). However, the relationship between nutritional status and the quality of bowel preparation in this population remains unexplored.

**Aims:**

This study aims to investigate the association between nutritional status at admission and inadequate bowel preparation in patients with IBD.

**Methods:**

This retrospective cohort study included 697 patients diagnosed with IBD at Chongqing General Hospital between 2021 and 2025. Nutritional status was comprehensively evaluated using three validated screening tools: the Prognostic Nutritional Index (PNI), the Nutritional Risk Index (NRI), and the Nutritional Risk Screening (NRS). Logistic regression analyses were performed to identify predictors of inadequate bowel preparation. Restricted cubic spline analysis was employed to examine potential nonlinear relationships, and subgroup analysis was performed to explore possible interactions. Furthermore, predictive performance was assessed and compared using receiver operating characteristic curves, net reclassification improvement, and integrated discrimination improvement.

**Results:**

The prevalence of malnutrition in patients with IBD, as assessed by the PNI, NRS, and NRI, was 6.3, 8.8, and 28.0%, respectively. Logistic regression analysis identified PNI, NRS, and NRI as independent risk factors for inadequate bowel preparation. Restricted cubic spline analysis revealed significant nonlinear relationships between inadequate bowel preparation and nutritional status, particularly as measured by PNI and NRI. Significant effects were observed in specific subgroups. Among the three indices, the NRI demonstrated the best predictive performance in this study.

**Conclusion:**

Nutritional status assessed at admission may be an independent predictor of inadequate bowel preparation in patients with inflammatory bowel disease.

## Introduction

Inflammatory bowel disease (IBD) comprises a group of heterogeneous, immune-mediated inflammatory disorders that affect the gastrointestinal tract, including ulcerative colitis (UC) and Crohn’ s disease (CD) (1, 2). Although the exact etiology of IBD remains incompletely understood, its chronic and relapsing nature, coupled with the high costs of treatment, significantly impairs patients’ quality of life (3, 4). Timely diagnosis and early assessment of disease activity are therefore crucial for reducing the therapeutic burden and improving patient outcomes (5, 6).

Endoscopic examination plays a pivotal role in the management of patients with IBD. Colonoscopy with biopsy is essential for establishing the diagnosis of IBD, conducting colorectal cancer (CRC) surveillance, evaluating endoscopic disease activity, detecting post-operative recurrence in CD, and assessing response to therapeutic interventions (7, 8). In particular, mucosal healing has emerged as a key treatment goal, as it is associated with sustained clinical remission, reduced risk of complications, and lower rates of hospitalization and surgical procedures (9). Adequate bowel preparation is fundamental to performing high-quality colonoscopy (10), a requirement that is especially critical in IBD patients, particularly in the context of monitoring malnutrition-related outcomes (11).

The estimated prevalence of malnutrition among patients with IBD ranges from 6.1 to 69.7% (12–16). Both malnutrition and sarcopenia are associated with adverse clinical outcomes, including higher hospitalization rates, suboptimal treatment responses, and diminished quality of life (12, 13, 17, 18). More than 70% of patients report that nutritional deficiencies considerably influence the disease course and exacerbate the frequency and severity of symptoms (19, 20). Currently, the Prognostic Nutritional Index, Nutritional Risk Index, and Nutritional Risk Screening are widely utilized for malnutrition screening (5, 6, 21). These indices can be derived from cost-effective and readily available parameters—such as serum albumin levels, total peripheral lymphocyte counts, and body weight—making them highly applicable in clinical study (21). Nevertheless, scant data exists on the relationship between these nutritional indices and the quality of bowel preparation in patients with IBD.

Therefore, this study aims to evaluate the prevalence of malnutrition among patients with IBD at the time of hospital admission using various nutritional screening tools—specifically PNI, NRI, and NRS—and to investigate their predictive capacity for inadequate bowel preparation (IBP).

## Methods

### Study design

This retrospective study was conducted at Chongqing General Hospital (Chongqing, China) between 2021 and 2025. The study protocol was reviewed and approved by the Medical Ethics Committee of Chongqing General Hospital (Approval No.: IIT S2025–035 - 01). All procedures performed in this study were in accordance with the ethical standards outlined in the Declaration of Helsinki.

### Study population

Patients were included in the study if they met the following criteria: (1) diagnosis of inflammatory bowel disease established based on clinical, endoscopic, radiological, and histological findings; (2) age 18 years or older; (3) completion of a colonoscopy with a comprehensive report; and (4) availability of a full nutritional assessment conducted on the first day of hospitalization. Patients with missing essential data—including height, weight, lymphocyte count, albumin level, or NRS score—on the first day of admission were excluded.

### Data extraction

The following data were retrospectively collected from the electronic medical records during the enrollment period:

(1) Demographic characteristics: sex, age, weight, height, smoking status, alcohol use, etc.; (2) Comorbidities: including chronic diseases, constipation, diarrhea, abdominal pain, and history of abdominal surgery; (3) Laboratory parameters: sodium, potassium, calcium, white blood cell count, red blood cell count, platelet count, serum albumin, lymphocyte count, hemoglobin, etc.; (4) Outcome: bowel preparation quality during hospitalization, as evaluated using the Boston Bowel Preparation Scale (BBPS).

For variables with repeated measurements, the first value recorded during hospitalization was used.

### Nutritional assessment

#### Nutritional risk index (NRI)

The Nutritional Risk Index (NRI) was calculated using the following formula:


$$\begin{array}{l} \mathrm{NRI}=1.519\times \text{serum albumin level}\;(g/L)+\\ 41.7\times (\begin{array}{l} \text{actual body weight}\;(\mathrm{kg})/\\ \text{ideal body weight}\;(\mathrm{kg}) \end{array}). \end{array}$$

Ideal body weight (kg) was determined based on gender-specific equations:


Formen:height(cm)–100–[(height–150)/4]

For women:height(cm)–100–[(height–150)/2.5]

In cases where the actual body weight exceeded the ideal body weight, the ratio of actual to ideal weight was set to 1 (21). A lower NRI value indicates poorer nutritional status. Based on their NRI scores, the enrolled patients were categorized into two groups: patients without nutritional risk (NRI ≥ 97.5) and patients with nutritional risk (NRI < 97.5).

#### Prognostic nutritional index (PNI)

The Prognostic Nutritional Index (PNI) was calculated using the following formula: PNI = serum albumin (g/L) + 5 × total lymphocyte count (10^9^/L). Based on their PNI scores, the enrolled patients were categorized into two groups: patients without nutritional risk (PNI ≥ 38) and patients with nutritional risk (PNI < 38).

#### Nutritional risk screening (NRS)

Nutritional risk was assessed using the NRS within 48 h after admission. This tool comprises three components: nutritional status (evaluated based on weight loss, body mass index [BMI], and food intake), disease severity (reflecting stress metabolism associated with the condition), and age (≥ 70 years). Total scores range from 0 to 7. A score of ≥ 3 was classified as “at nutritional risk,” while a score below 3 indicated “no nutritional risk.” Body weight and height were measured by nurses, whereas attending physicians documented food intake and weight loss. Nutritional screening was initially performed by trained nurses and subsequently reviewed by clinical dietitians.

### Outcome and assessment

The primary outcome of this study was the quality of bowel preparation, evaluated using the Boston Bowel Preparation Scale (BBPS). Bowel preparation was considered adequate if all three segmental BBPS scores were ≥ 2, as assessed after cleaning maneuvers (22, 23). Each segment of the colon—left, transverse, and right—was assessed using the Boston Bowel Preparation Scale (BBPS) and assigned a score ranging from 0 (inadequate) to 3 (excellent). Following cleansing maneuvers, the scores were determined as follows:

0: Mucosa not visible due to presence of solid stool that could not be cleared;

1: Partial mucosal visualization with some areas obscured by staining, residual stool, and/or opaque liquid;

2: Minor residual staining, but full mucosal visualization achieved;

3: Entire mucosa of the segment clearly visible.

Anatomically, the right colon encompassed the cecum and ascending colon; the left colon included the descending colon, sigmoid colon, and rectum; and the transverse colon segment comprised the hepatic and splenic flexures. The overall BBPS score was calculated as the sum of the scores from all three segments, ranging from 0 (completely unprepared) to 9 (excellent).

### Statistical analysis

This study was reported in accordance with the recommendations of the Strengthening the Reporting of Observational Studies in Epidemiology (STROBE) statement (24). First, statistical descriptions and intergroup comparisons were performed. The normality of continuous variables was assessed using the Shapiro–Wilk test, which indicated that most variables were not normally distributed. Continuous variables were summarized as medians with interquartile ranges, while categorical variables were expressed as frequencies and percentages. Comparisons between groups for continuous variables were conducted using Mann - Whitney U test, whereas categorical variables were compared using either the chi-square test or Fisher’s exact test, as appropriate.

Logistic regression analyses were performed to evaluate the association between nutritional status at admission and inadequate bowel preparation. Odds ratios (ORs) along with their corresponding 95% confidence intervals (CIs) were calculated. All regression models were constructed with three sequential levels of adjustment: Model 1 was unadjusted; Model 2 was adjusted for age, sex, smoking status, and drinking status; and Model 3 included all covariates from Model 2, with additional adjustment for constipation, diarrhea, abdominal pain, perianal lesions, history of inadequate bowel preparation, history of abdominal surgery, use of antidepressants, and biological therapy. To mitigate potential multicollinearity, albumin, lymphocytes, and body mass index (BMI) were not incorporated into the models. Collinearity among continuous variables was assessed using the variance inflation factor (VIF), and variables with a VIF exceeding 5 were excluded from the final model.

Restricted cubic spline (RCS) is a widely used method for characterizing potential nonlinear dose–response relationships between continuous exposures and outcomes. The approach partitions the observed range of a continuous variable into k segments defined by knots, typically placed at fixed percentiles of the distribution. Within each segment, a cubic polynomial is applied, ensuring smoothness at the knots while constraining the ends to be linear beyond the first and last knots (21). In this study, RCS models were employed to visualize the association of the Nutritional Risk Index (NRI) and the Prognostic Nutritional Index (PNI) with the risk of inadequate bowel preparation. The variables included in the RCS models were consistent with those specified in Model 3.

In addition, subgroup analyses were conducted to further evaluate the associations of the NRI, PNI, and NRS with inadequate bowel preparation across different subpopulations. The subgroups were stratified based on the following criteria: age (< 40 years vs. ≥ 40 years), sex (male vs. female), and the presence of intestinal stricture (yes vs. no).

Finally, to evaluate the incremental predictive value of NRI, PNI, and NRS for inadequate bowel preparation, the area under the receiver operating characteristic curve (AUROC) was used to quantify the discriminative ability of each model. The DeLong method was subsequently applied to assess the significance of changes in AUC after the inclusion of NRI, PNI, and NRS. Next, the net reclassification improvement and integrated discrimination improvement indices were calculated to assess the risk reclassification ability of NRI, PNI, and NRS. Lastly, a chi-square likelihood ratio test was performed to determine whether models incorporating NRI, PNI, and NRS offered a significantly better fit compared to those without these predictors. Statistical analysis was performed using R software (version 4.3.1, https://www.r-project.org/). A P - value < 0.05 was considered statistically significant.

## Results

### Baseline characteristics of participants

**Figure 1:**
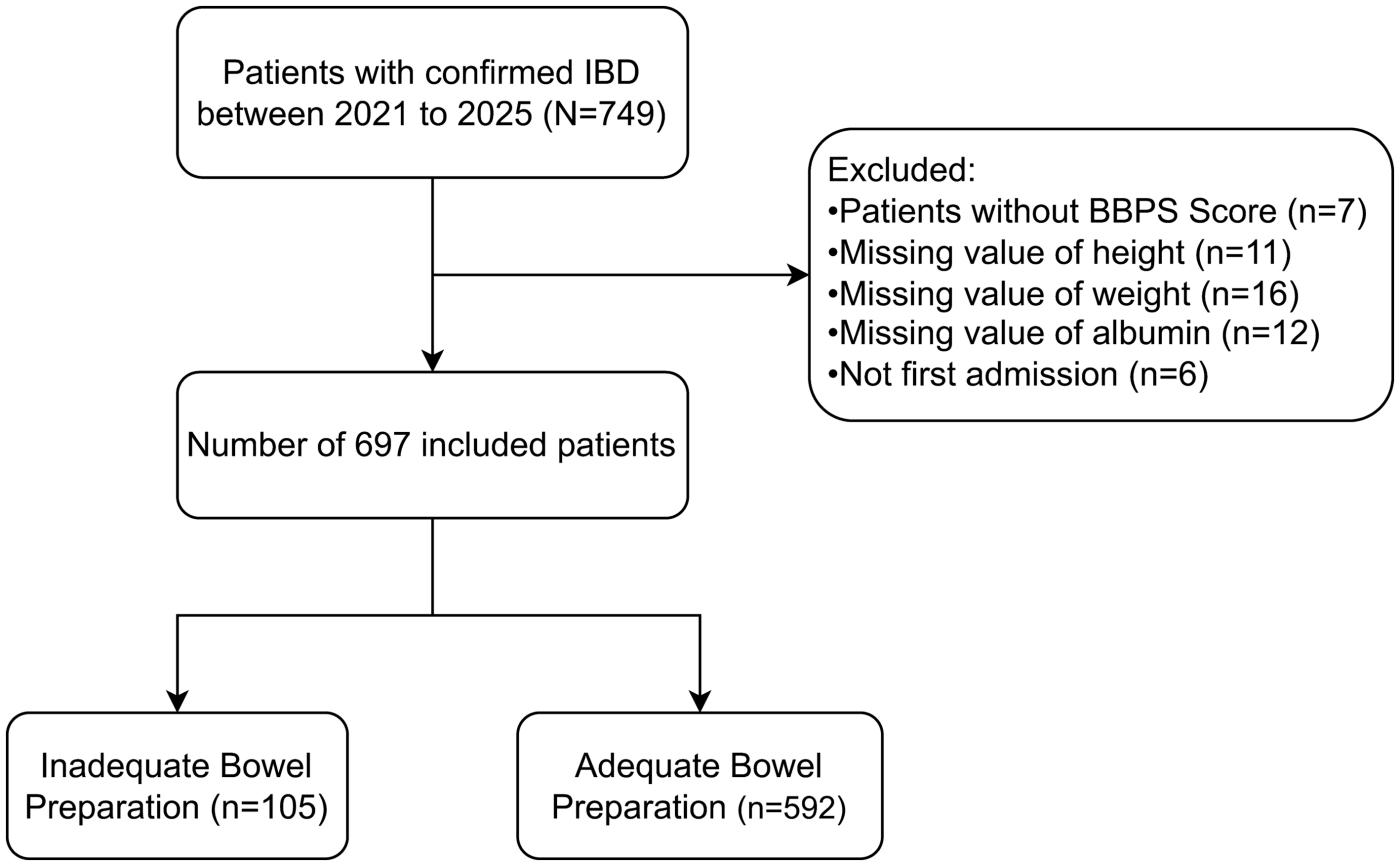
Flow chart of study population.

A total of 749 hospitalized patients diagnosed with inflammatory bowel disease were initially enrolled in this study. After exclusions, 697 patients were included in the final analysis (Figure 1). The demographic and clinical characteristics of the participants are presented in Table 1. The overall prevalence of inadequate bowel preparation was 15.1%. Among the included patients, 451 (64.7%) were male, with a median age of 29 (22–42) years. The median disease duration of IBD was 2.00 (1.00–5.00) years. Additionally, 54 (7.7%) patients had a history of smoking, 34 (4.9%) had a history of alcohol use, 112 (16.1%) had a history of abdominal surgery, and 124 (17.8%) had a history of inadequate bowel preparation.

**Table 1:** The demographic and clinical characteristics of the IBD patients.

Variables	Overall (*n* = 697)	Adequate bowel preparation (*n* = 592)	Inadequate bowel preparation (*n* = 105)	*p*-value
Demographic				
Age, years	29.00 (22.00, 42.00)	29.00 (22.00, 39.00)	32.00 (24.00, 57.00)	0.002
Sex (%)				0.975
Male	451 (64.7)	383 (64.7)	68 (64.8)	
Female	246 (35.3)	209 (35.3)	37 (35.2)	
Years of diagnosis (years)	2.00 (1.00, 5.00)	2.00 (1.00, 5.00)	3.00 (1.00, 6.00)	0.655
BMI (kg/m^2^)	20.20 (18.31, 22.49)	20.20 (18.28, 22.41)	20.20 (18.72, 22.49)	0.716
Insurance (%)				0.046
Residents ‘medical insurance	448 (64.3)	371 (62.7)	77 (73.3)	
Employee medical insurance	249 (35.7)	221 (37.3)	28 (26.7)	
Education (%)				0.126
High school and below	347 (49.8)	287 (48.5)	60 (57.1)	
College or above	350 (50.2)	305 (51.5)	45 (42.9)	
Marital status (%)				0.055
Married	357 (51.2)	303 (51.2)	54 (51.4)	
Unmarried	327 (46.9)	281 (47.5)	46 (43.8)	
Others	13 (1.9)	8 (1.4)	5 (4.8)	
Type (%)				0.168
UC	214 (30.7)	175 (29.6)	39 (37.1)	
CD	410 (58.8)	357 (60.3)	53 (50.5)	
IBD-U	73 (10.5)	60 (10.1)	13 (12.4)	
Smoking (%)				0.589
Yes	54 (7.7)	44 (7.4)	10 (9.5)	
No	643 (92.3)	548 (92.6)	95 (90.5)	
Drinking (%)				0.097
Yes	34 (4.9)	25 (4.2)	9 (8.6)	
No	663 (95.1)	567 (95.8)	96 (91.4)	
Laboratory parameters				
White blood cell (10^9^/L)	6.07 (4.96, 7.88)	5.99 (4.89, 7.62)	6.84 (5.41, 8.84)	0.002
Red blood cell (10^9^/L)	4.72 (4.20, 5.14)	4.78 (4.25, 5.18)	4.40 (3.92, 4.88)	<0.001
Hemoglobin (g/dL)	134.00 (116.00, 148.00)	136.00 (117.00, 149.00)	127.00 (109.00, 141.00)	0.001
Platelet (10^9^/L)	267.00 (216.00, 350.00)	263.00 (215.00, 340.25)	292.00 (228.00, 407.00)	0.016
Neutrophils (10^9^/L)	4.03 (2.95, 5.55)	3.96 (2.90, 5.37)	4.85 (3.42, 6.65)	<0.001
Lymphocytes (10^9^/L)	1.54 (1.20, 1.92)	1.50 (1.16, 1.90)	1.69 (1.37, 1.99)	0.001
C-reactive protein (mg/L)	4.47 (1.04, 16.75)	3.84 (0.97, 12.86)	15.01 (2.73, 40.99)	<0.001
Albumin (g/L)	42.80 (39.20, 45.90)	43.40 (39.90, 46.10)	40.60 (36.10, 43.80)	<0.001
ESR (mm/h)	15.00 (6.00, 29.00)	14.00 (6.00, 26.00)	21.00 (9.00, 42.00)	<0.001
Sodium (mEq/L)	139.30 (137.90, 140.80)	139.30 (137.90, 140.80)	138.60 (137.40, 140.70)	0.049
Potassium (mEq/L)	3.96 (3.75, 4.18)	3.97 (3.77, 4.18)	3.87 (3.58, 4.16)	0.011
Calcium (mmol/L)	2.35 (2.27, 2.44)	2.36 (2.29, 2.45)	2.31 (2.20, 2.38)	<0.001
Comorbidities				
Constipation (%)				0.349
Yes	5 (0.7)	3 (0.5)	2 (1.9)	
No	692 (99.3)	589 (99.5)	103 (98.1)	
Diarrhea (%)				0.161
Yes	351 (50.4)	291 (49.2)	60 (57.1)	
No	346 (49.6)	301 (50.8)	45 (42.9)	
Abdominal pain (%)				0.707
Yes	350 (50.2)	295 (49.8)	55 (52.4)	
No	347 (49.8)	297 (50.2)	50 (47.6)	
Perianal lesion (%)				0.955
Yes	234 (33.6)	198 (33.4)	36 (34.3)	
No	463 (66.4)	394 (66.6)	69 (65.7)	
History of Inadequate bowel preparation (%)				<0.001
Yes	124 (17.8)	75 (12.7)	49 (46.7)	
No	573 (82.2)	517 (87.3)	56 (53.3)	
Family history of colorectal cancer (%)				0.328
Yes	1 (0.1)	0 (0.0)	1 (1.0)	
No	696 (99.9)	592 (100.0)	104 (99.0)	
History of abdominal pelvic surgery (%)				0.449
Yes	112 (16.1)	92 (15.5)	20 (19.0)	
No	585 (83.9)	500 (84.5)	85 (81.0)	
Antidepressants (%)				0.967
Yes	6 (0.9)	5 (0.8)	1 (1.0)	
No	691 (99.1)	587 (99.2)	104 (99.0)	
NRI (%)				<0.001
No risk group	502 (72.0)	445 (75.2)	57 (54.3)	
Risk group	195 (28.0)	147 (24.8)	48 (45.7)	
PNI (%)				0.001
No risk group	653 (93.7)	563 (95.1)	90 (85.7)	
Risk group	44 (6.3)	29 (4.9)	15 (14.3)	
NRS (%)				0.018
No risk group	636 (91.2)	547 (92.4)	89 (84.4)	
Risk group	61 (8.8)	45 (7.6)	16 (15.2)	
Biological (%)				0.234
Upadacitinib	5 (0.7)	5 (0.8)	0 (0.0)	
Ustekinumab	85 (12.2)	79 (13.3)	6 (5.7)	
Infliximab	227 (32.6)	192 (32.4)	35 (33.3)	
Vedolizumab	57 (8.2)	49 (8.3)	8 (7.6)	
Adalimumab	66 (9.5)	56 (9.5)	10 (9.5)	
None	257 (36.9)	211 (35.6)	46 (43.8)	
State of disease (%)				0.001
Active period	380 (54.5)	306 (51.7)	74 (70.5)	
Inactive period	317 (45.5)	286 (48.3)	31 (29.5)	
Intestinal infection (%)				0.003
Yes	114 (16.4)	86 (14.5)	28 (26.7)	
No	583 (83.6)	506 (85.5)	77 (73.3)	
Enteral nutrition (%)				0.973
Yes	316 (45.3)	268 (45.3)	48 (45.7)	
No	381 (54.7)	324 (54.7)	57 (54.3)	
Intestinal stricture (%)				0.043
Yes	127 (18.2)	100 (16.9)	27 (25.7)	
No	570 (81.8)	492 (83.1)	78 (74.3)	
Interval time (%)				0.331
Yes	238 (34.1)	207 (35.0)	31 (29.5)	
No	459 (65.9)	385 (65.0)	74 (70.5)	
Chronic diseases (%)				0.345
Yes	139 (19.9)	114 (19.3)	25 (23.8)	
No	558 (80.1)	478 (80.7)	80 (76.2)	
Scores				
Boston (score)	7.00 (6.00, 9.00)	7.00 (7.00, 9.00)	3.00 (0.00, 5.00)	<0.001
LC (score)	3.00 (2.00, 3.00)	3.00 (2.00, 3.00)	1.00 (0.00, 1.00)	<0.001
TC (score)	2.00 (2.00, 3.00)	2.00 (2.00, 3.00)	1.00 (0.00, 2.00)	<0.001
RC (score)	2.00 (2.00, 3.00)	2.00 (2.00, 3.00)	1.00 (0.00, 2.00)	<0.001
NRS-2002 (score)	3.00 (1.00, 3.00)	3.00 (1.00, 3.00)	3.00 (0.00, 3.00)	0.295
Braden (score)	23.00 (22.00, 23.00)	23.00 (22.00, 23.00)	23.00 (22.00, 23.00)	0.261

Continuous variables were presented as median (interquartile range) and categorical variables as number (percentage). The Kruskal-Wallis test was used to compare group differences for continuous variables, and the Chi-squared test was used to compare group differences for categorical variables. BMI: Body mass index; UC: ulcerative colitis; CD: Crohn’ s disease; IBD-U: inflammatory bowel disease unclassified; ESR: Erythrocyte sedimentation Rate; NRI: Nutritional Risk Index; PNI: Prognostic Nutritional Index; NRS-2002: Nutritional Risk Screening 2002; LC: Left colon; TC: Transverse colon; RC: right colon.

### Nutritional status and inadequate bowel preparation

The prevalence of malnutrition, as assessed by the Nutritional Risk Index, was 28%; by the Prognostic Nutritional Index, 6.3%; and by the NRS, 8.8%. As presented in Table 2, significant associations were observed between nutritional status—evaluated using NRI, PNI, and NRS—and inadequate bowel preparation (IBP). Logistic regression analysis revealed that patients in the nutritional risk group had a significantly higher risk of IBP compared to those in the non-nutritional risk group. After adjusting for confounding factors in Models 2 and 3, the nutritional risk group continued to exhibit a significantly increased risk of IBP relative to the non-risk group (all *p* < 0.05; Table 2).

**Table 2:** Logistic Regression: association between nutrition risk group and bowel preparation.

Variables	Model 1		Model 2		Model 3	
	Unadjusted OR (95% CI)	*P*-value	Adjusted OR (95% CI)	*P*-value	Adjusted OR (95% CI)	*P*-value
NRI	2.55 (1.66, 3.91)	<0.001	2.52 (1.62, 3.89)	<0.001	2.07 (1.26, 3.41)	<0.001
PNI	3.24 (1.63, 6.19)	<0.001	2.49 (1.19, 4.97)	0.001	2.46 (1.21, 5.35)	0.001
NRS	2.18 (1.15, 3.96)	0.002	2.04 (1.06, 3.76)	0.026	2.07 (1.02, 4.06)	0.037

NRI: Nutritional Risk Index; PNI: Prognostic Nutritional Index; NRS: Nutritional Risk Screening 2002; OR: odds ratio, CI: confidence interval. Model 2, adjusted for age, sex, smoking, and drinking. Model 3, adjusted for model 2 plus Constipation, Diarrhea, Abdominal pain, Perianal lesion, Inadequate bowel preparation, History of abdominal surgery, Antidepressants, Biological, State of Disease, Intestinal infection and Intestinal stricture.

### Restricted cubic spline

**Figure 2:**
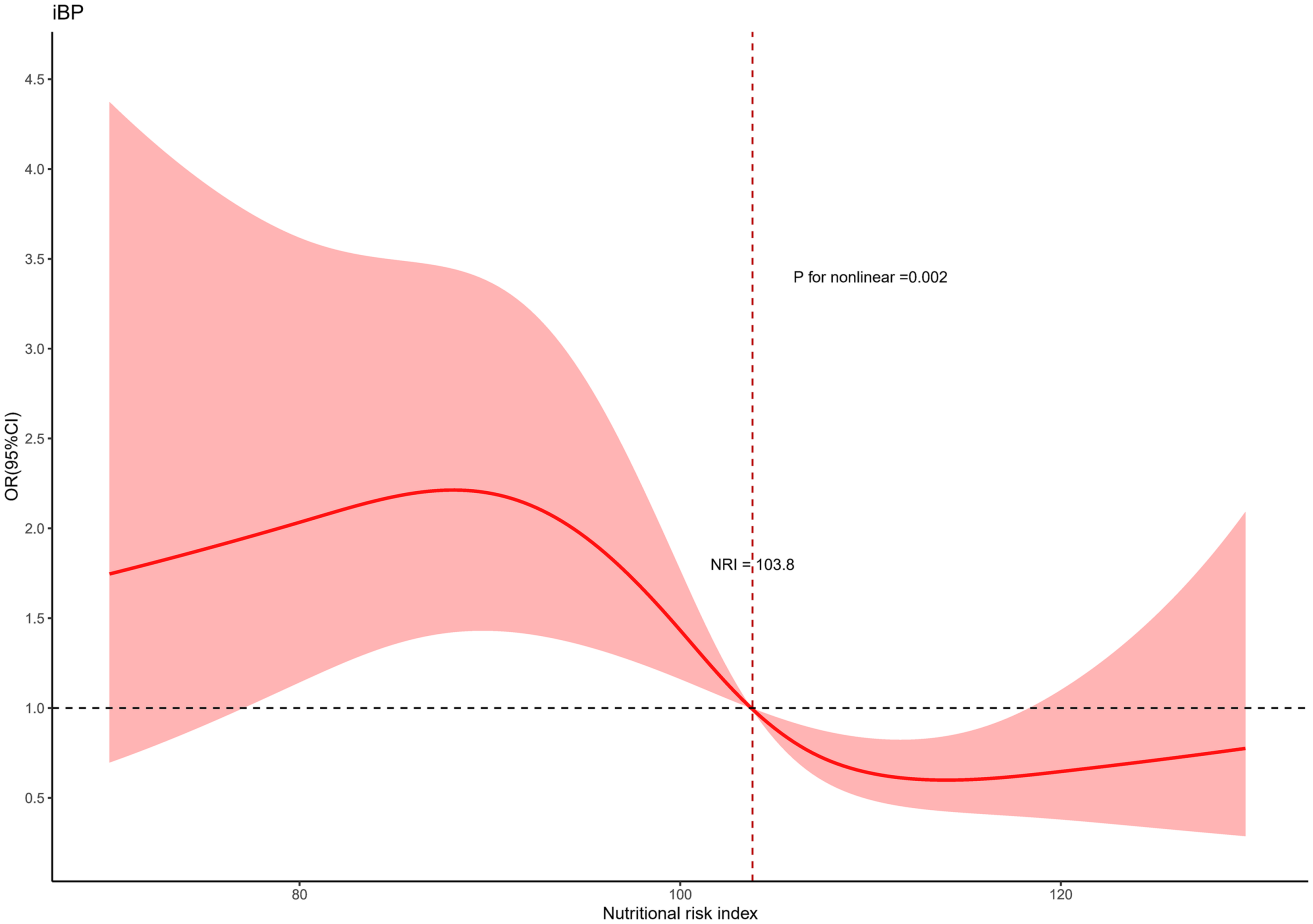
Restricted spline curves for the associations between the nutritional risk index and IBP.

**Figure 3:**
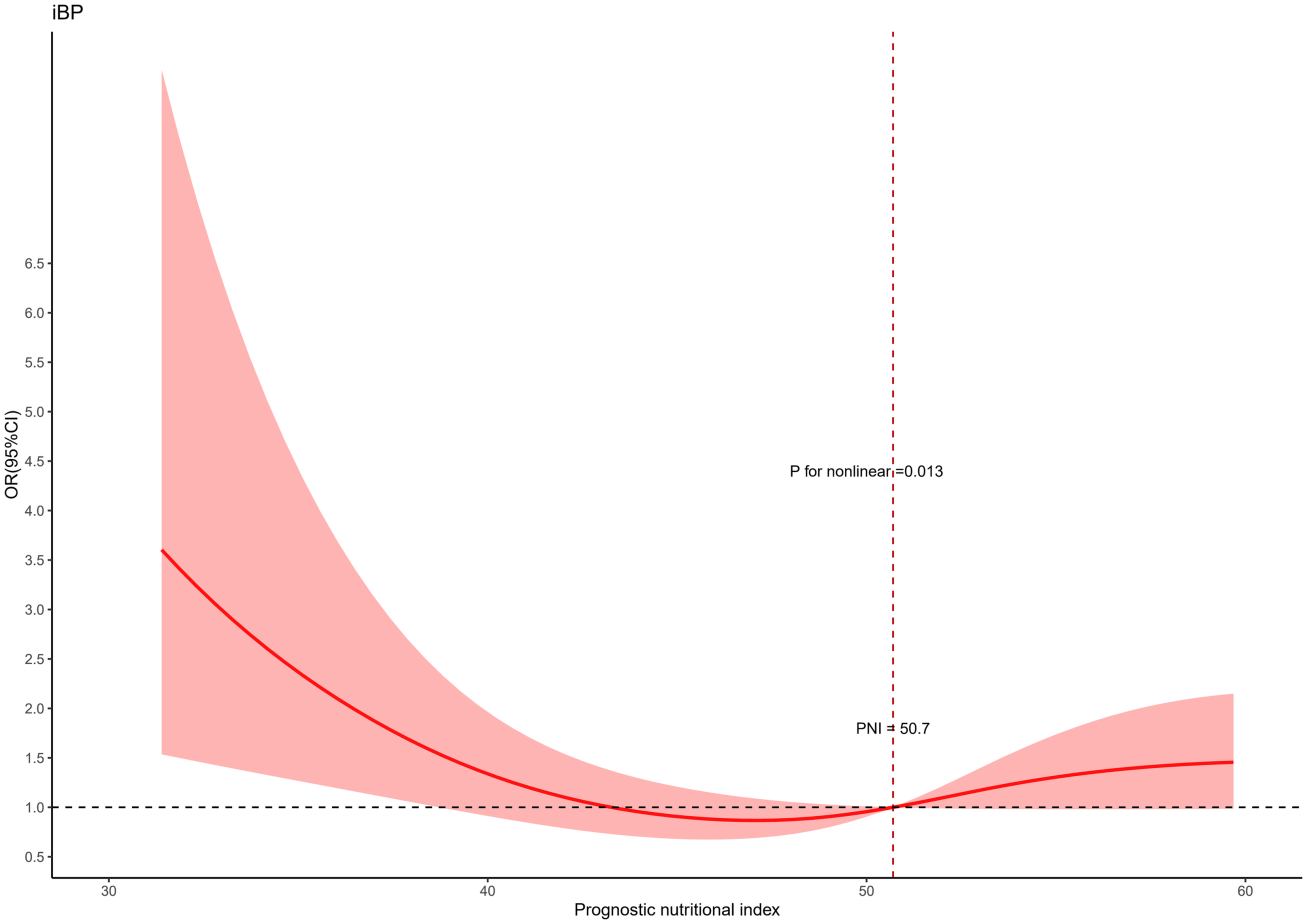
Restricted spline curves for the associations between the prognostic nutritional index and IBP.

Restricted cubic splines were employed to further investigate the associations of the NRI and the PNI with inadequate bowel preparation (IBP). Both NRI and PNI were analyzed as continuous variables and were adjusted for confounders using Model 3. The NRS was excluded from this analysis, as it could not be treated as a continuous variable. As illustrated in Figures 2, 3, nonlinear relationships were identified between IBP and both NRI and PNI.

### Subgroup analysis

**Figure 4:**
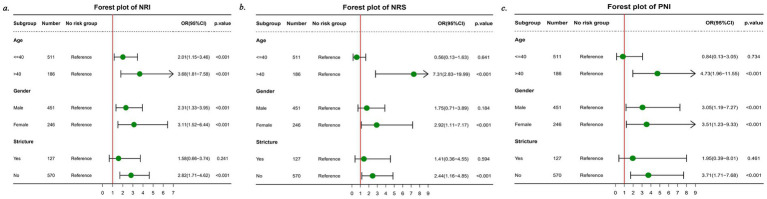
Forest plot for subgroup analyses.

A subgroup analysis was performed to assess the associations of the NRI, PNI, and NRS with inadequate bowel preparation (IBP) in different subpopulations (Figure 4). The results revealed inconsistent associations between NRI and IBP in the intestinal stricture subgroup, inconsistent associations of PNI with IBP in both the age and intestinal stricture subgroups, and inconsistent associations of NRS with IBP across all three subgroups.

### Additive value of NRI in IBP risk prediction

The predictive performance of each model was assessed using the area under the curve (AUC). The addition of NRI, PNI, and NRS to the base model led to a modest increase in AUC values; however, only the increase associated with NRI reached statistical significance (Table 3). To evaluate the risk reclassification capability of the models, net reclassification improvement and integrated discrimination improvement were computed. The inclusion of NRI, PNI, and NRS resulted in significant increases in both the net reclassification improvement and integrated discrimination improvement indices for most models (all *p* < 0.05; Table 3), suggesting that these indices may enhance the risk reclassification performance of the prediction models. Furthermore, likelihood ratio tests were conducted. For Models 2 and 3, the incorporation of NRI significantly improved the accuracy of IBP prediction, while the addition of PNI and NRS also contributed to significantly better risk reclassification.

**Table 3:** Discrimination of each predictive model for bowel preparation.

Predictive models	AUC		Net reclassification improvement		Integrated discrimination improvement		Likelihood ratio test
	Estimate (95% CI)	*P*-value	Estimate (95% CI)	*P*-value	Estimate (95% CI)	*P*-value	*P*-value
Model 2 without NRI	0.598 (0.535, 0.664)						
Model 2 with NRI	0.652 (0.589, 0.721)	0.004	0.418 (0.215, 0.621)	<0.001	0.029 (0.014, 0.043)	<0.001	<0.001
Model 3 without NRI	0.721 (0.667, 0.779)						
Model 3 with NRI	0.751 (0.698, 0.801)	<0.001	0.421 (0.218, 0.634)	<0.001	0.019 (0.006, 0.011)	0.007	<0.001
Model 2 without PNI	0.598 (0.533, 0.662)						
Model 2 with PNI	0.602 (0.534, 0.660)	0.693	0.155 (0.000, 0.311)	0.05	0.013 (0.001, 0.024)	0.029	0.016
Model 3 without PNI	0.725 (0.666, 0.778)						
Model 3 with PNI	0.726 (0.667, 0.781)	0.881	0.141 (−0.046, 0.328)	0.139	0.015 (0.002, 0.028)	0.024	0.014
Model 2 without NRS	0.593 (0.526, 0.662)						
Model 2 with NRS	0.598 (0.528, 0.654)	0.71	0.152 (0.008, 0.297)	0.037	0.010 (0.001, 0.019)	0.029	0.033
Model 3 without NRS	0.720 (0.660, 0.778)						
Model 3 with NRS	0.725 (0.666, 0.777)	0.386	0.235 (0.075, 0.394)	0.003	0.012 (0.001, 0.022)	0.031	0.028

AUC: area under curve; NRI: Nutritional Risk Index; PNI: Prognostic Nutritional Index; NRS: Nutritional Risk Screening 2002; OR: odds ratio, CI: confidence interval. Model 2, adjusted for age, sex, smoking, and drinking. Model 3, adjusted for model 2 plus Constipation, Diarrhea, Abdominal pain, Perianal lesion, Inadequate bowel preparation, History of abdominal surgery, Antidepressants, Biological.

## Discussion

### Main findings

To our knowledge, there are no previous studies investigating the association between nutritional status and inadequate bowel preparation in patients with inflammatory bowel disease (IBD). This study is the first to demonstrate that the nutritional status of IBD patients at admission, as assessed by the Nutritional Risk Index (NRI), Prognostic Nutritional Index (PNI), and Nutritional Risk Screening (NRS), may be associated with inadequate bowel preparation. In this study, compared to the non-nutritional risk group, the nutritional risk group showed a significantly higher risk of inadequate bowel preparation, suggesting that nutritional status at admission may serve as an independent risk factor for inadequate bowel preparation in IBD patients. Furthermore, we evaluated the additional predictive value of NRI, PNI, and NRS for the risk of inadequate bowel preparation. The results indicated that incorporating NRI into the prediction model may enhance both its accuracy and risk reclassification ability. In contrast, PNI and NRS improved only the risk reclassification ability of the model.

### Possible explanations for the study results

Malnutrition may increase the risk of inadequate bowel preparation in patients with IBD; however, the underlying pathophysiological mechanisms remain incompletely elucidated. Existing evidence suggests several potential explanations. Firstly, reduced oral food intake is a key contributor to malnutrition in IBD patients. These individuals are often advised to—or voluntarily adopt—a low-residue or low-fiber diet. Stool lacking sufficient fiber tends to be more viscous and adherent to the intestinal mucosa, thereby impeding effective clearance during bowel preparation. Furthermore, the inflammatory state of the intestine exacerbates this condition. Second, malnutrition can result in the atrophy and weakening of intestinal smooth muscles. Intestinal peristalsis, which serves as the propulsive force for moving contents through the digestive tract, becomes slower and less efficient when the muscles are compromised. As a result, the weakened peristaltic activity is insufficient to effectively propel laxatives or intestinal contents toward the distal end for excretion. In addition, malnutrition—such as deficiencies in trace elements (e.g., zinc and selenium) and vitamin D—can compromise mucosal barrier integrity and immune regulatory function, potentially exacerbating intestinal inflammation. Finally, malnourished patients often experience generalized weakness and fatigue, which may reduce their tolerance for the large volume of fluids required with laxative use. As a result, they are more likely to discontinue or reduce treatment prematurely due to discomfort, such as nausea, vomiting, or abdominal distension.

### Implications for clinical practice

The primary therapeutic objective in IBD is the achievement of endoscopic remission, underscoring the critical importance of adequate bowel preparation. Inadequate bowel preparation not only increases the burden on patients but also leads to unnecessary resource utilization. This study identified malnutrition at the time of admission may be an independent risk factor for inadequate bowel preparation in patients with IBD. This finding holds potential clinical relevance, particularly for the management of IBD patients presenting with nutritional risk who require bowel preparation. First, it emphasizes the close association between the nutritional status of IBD patients upon hospital admission and the risk of inadequate bowel preparation. This highlights the critical importance of conducting a rigorous assessment of nutritional status at the time of admission. Consequently, when evaluating the risk of inadequate bowel preparation in IBD patients, clinical healthcare providers should pay special attention to assessing nutritional status and implement proactive, effective bowel preparation strategies to enhance its quality. Second, this study demonstrates that the NRI, as a simple indicator, may play a critical role in assessing the risk of inadequate bowel preparation among patients with IBD. Integrating the NRI into predictive models could substantially improve their performance, thereby offering valuable guidance for clinical healthcare providers to more accurately identify IBD patients susceptible to inadequate bowel preparation and to initiate appropriate interventions accordingly. Additionally, this study emphasizes the role of NRI, PNI, and NRS in improving risk reclassification. Incorporating these indices into clinical models allows for more accurate risk stratification of patients, thereby enhancing the identification of high-risk individuals and supporting the implementation of targeted interventions. However, it is important for clinical healthcare providers to recognize that NRI, PNI, and NRS cannot comprehensively capture the full spectrum of malnutrition severity, as they omit specific factors such as vitamin deficiencies and sarcopenia. Finally, discrepancies were observed in the subgroup of IBD patients with intestinal strictures. The small sample size in this subgroup may have introduced bias and reduced the statistical power, potentially obscuring the associations between NRI, PNI, NRS, and inadequate bowel preparation. Therefore, when evaluating the risk of inadequate bowel preparation in patients with IBD, clinicians should consider the presence of intestinal strictures and interpret admission nutrition assessments with caution.

### Limitations

This study has some limitations. First, this was a single-center retrospective study. Due to the retrospective design, causality cannot be inferred. Prospective studies are warranted to further clarify the relationship between nutritional status and inadequate bowel preparation in patients with inflammatory bowel disease (IBD). Second, nutritional status was assessed only at the time of admission, which does not account for potential dynamic changes over time. Finally, although multivariable adjustments and subgroup analyses were performed, residual confounding from unmeasured factors may still have influenced the results.

## Conclusion

In conclusion, this study confirms the association between nutritional status at admission, as assessed by NRI, PNI, and NRS, and inadequate bowel preparation in patients with IBD. These findings hold significant clinical relevance for predicting and preventing inadequate bowel preparation in this patient population. Future clinical practice and research should build upon these results to further elucidate the underlying mechanisms and develop effective interventions targeting nutritional status to improve bowel preparation outcomes in IBD patients.

## Data Availability

The raw data supporting the conclusions of this article will be made available by the authors, without undue reservation.
